# Utilization of nanopore direct RNA sequencing to analyze viral RNA modifications

**DOI:** 10.1128/msystems.01163-23

**Published:** 2024-01-31

**Authors:** Lu Tan, Zhihao Guo, Xiaoming Wang, Dal Young Kim, Runsheng Li

**Affiliations:** 1 Department of Infectious Diseases and Public Health, Jockey Club College of Veterinary Medicine and Life Sciences, City University of Hong Kong, Hong Kong, China; 2 College of Veterinary Medicine, Nanjing Agricultural University, Nanjing, China; 3 Tung Biomedical Sciences Centre, City University of Hong Kong, Hong Kong, China; Génomique Métabolique, Genoscope, Institut François Jacob, CEA, CNRS, Université Évry, Université Paris-Saclay, Évry-Courcouronnes, France

**Keywords:** RNA modification, nanopore, comparative methods, alphavirus, Sindbis virus

## Abstract

Modifications on viral RNAs (vRNAs), either genomic RNAs or RNA transcripts, have complex effects on the viral life cycle and cellular responses to viral infection. The advent of Oxford Nanopore Technologies Direct RNA Sequencing provides a new strategy for studying RNA modifications. To this end, multiple computational tools have been developed, but a systemic evaluation of their performance in mapping vRNA modifications is lacking. Here, 10 computational tools were tested using the Sindbis virus (SINV) RNAs isolated from infected mammalian (BHK-21) or mosquito (C6/36) cells, with *in vitro*-transcribed RNAs serving as modification-free control. Three single-mode approaches were shown to be inapplicable in the viral context, and three out of seven comparative methods required cutoff adjustments to reduce false-positive predictions. Utilizing optimized cutoffs, an integrated analysis of comparative tools suggested that the intersected predictions of Tombo_com and xPore were significantly enriched compared with the background. Consequently, a pipeline integrating Tombo_com and xPore was proposed for vRNA modification detection; the performance of which was supported by N^6^-methyladenosine prediction in severe acute respiratory syndrome coronavirus 2 RNAs using publicly available data. When applied to SINV RNAs, this pipeline revealed more intensive modifications in subgenomic RNAs than in genomic RNAs. Modified uridines were frequently identified, exhibiting substantive overlapping between vRNAs generated in different cell lines. On the other hand, the interpretation of other modifications remained unclear, underlining the limitations of the current computational tools despite their notable potential.

**IMPORTANCE:**

Computational approaches utilizing Oxford Nanopore Technologies Direct RNA Sequencing data were almost exclusively designed to map eukaryotic epitranscriptomes. Therefore, extra caution must be exercised when using these tools to detect vRNA modifications, as in most cases, vRNA modification profiles should be regarded as unknown epitranscriptomes without prior knowledge. Here, we comprehensively evaluated the performance of 10 computational tools in detecting vRNA modification sites. All tested single-mode methods failed to differentiate native and *in vitro-*transcribed samples. Using optimized cutoff values, seven tested comparative tools generated very different predictions. An integrated analysis showed significant enrichment of Tombo_com and xPore predictions against the background. A pipeline for vRNA modification detection was proposed accordingly and applied to Sindbis virus RNAs. In conclusion, our study underscores the need for the careful application of computational tools to analyze viral epitranscriptomics. It also offers insights into alphaviral RNA modifications, although further validation is required.

## INTRODUCTION

Chemical modification of ribonucleotides is prevalent across all types of RNA molecules, and more than 150 residue alterations have been reported (1). These modifications are crucial for RNA metabolism and interactions with other molecules (2–5). Additionally, N^6^-methyladenosine (m6A) is the most abundant internal decoration in eukaryotic mRNAs and is the most widely studied RNA modification to date (6). It is also broadly reported in viral RNAs (vRNAs), either genomic RNAs (gRNAs) of RNA viruses or mRNAs transcribed from DNA viruses, RNA viruses, and retroviruses (7). It poses a complex impact on the viral life cycle and cellular response to viral infections, and both proviral and antiviral effects have been reported (7, 8). Other modifications, including pseudouridine, 5-methylcytidine (m5C), 2′-O-methylation (Nm), N^6^,2′-O-dimethyladenosine (m6Am), N^1^-methylguanosine (m1G), and N^4^-acetylcytidine (ac4C), are found to decorate vRNAs as well (9–11). Determining the location and stoichiometry of various RNA modifications on vRNAs is crucial for studying viral biology.

There are different RNA modification detecting approaches. Liquid chromatography-mass spectrometry (LC-MS) and nuclear magnetic resonance (NMR) are the most fundamental techniques (9, 12). They can detect almost all chemical modifications but do not allow the determination of sequence positions and are limited by the difficulty of purifying a particular RNA molecule (11). The development of several high-throughput approaches based on short-read sequencing enables transcriptome-wide mapping of modified ribonucleotides, in most cases, the m6A residuals (9). These methods can be antibody dependent, with the RNA fragments containing the modifications immunopurified with modification-specific antibodies, such as methylated RNA immunoprecipitation sequencing (MeRIP-Seq) (13) and m6A individual-nucleotide-resolution cross-linking and immunoprecipitation sequencing (MiCLIP-Seq) (14). Orthologous antibody-independent methodologies apply chemical or enzyme reactions to enrich the modifications (15–19). However, these approaches are limited by the biases and nonbiological variations associated with short-read sequencing (9). Moreover, given the compact nature of viral genomes, it is challenging for these short-read methods to distinguish the complex overlapping sequences of viral transcriptomes.

The advent of Oxford Nanopore Technologies (ONT) Direct RNA Sequencing (DRS) opened another avenue for studying epitranscriptomics while bypassing the limitations of short-read sequencing approaches. It records the ionic current changes as the full-length native RNA molecules pass through the nanopores, which can be converted into nucleotide sequences by a base caller. In many cases, modified ribonucleotides have a remarkable effect on the ionic signals and have direct impact on the accuracy of downstream base calling (9). Based on this principle, a bunch of computational tools have been developed to identify modified RNA sites by exploiting changes at the signal level or increased error rate (20).

A few software packages utilize trained models to identify RNA modifications, such as m6Anet (21) and Nanom6A (22). Both methods specifically recognize m6A sites within the DRACH/RRACH motif, the most preferred consensus sequence for eukaryotic m6A modification (23). Alternately, Tombo_de novo detects modified sites by performing a hypothesis test against the canonical model (unmodified bases) based on the reference genome sequence (24). Given the characteristic of running without a control sample, the above-mentioned computational methods are classified as single-mode tools. In contrast, several detection methods, referred to as comparative tools, evaluate the differences between the samples of interest and those lacking modifications to infer the modified nucleotides. For instance, m6A sites can be identified by comparing RNAs in untreated and METTL3 (a highly conserved m6A writer in eukaryotes) knockout cells (25). On the other hand, comparisons between wild-type and *in vitro*-transcribed (IVT) RNAs generated a full list of potentially modified sites without defining the modification types (26). Some comparative methods apply current signal features, such as median, mean, standard deviation, and dwell time, as inputs, including Nanocompore (27), Tombo_com (24), and xPore (28). Other tools use base calling errors, which may represent mismatch, indel, and base quality score variations. ELIGOS2 (29), EpiNano_Error (30), Differr (31), and DRUMMER (32) fall into this category. The characteristics of different computational methods are summarized in Table 1.

**TABLE 1:** Characteristics of computational tools used in the present study for ONT DRS-based RNA modification detection

Type and tool	Analysis type	Approach^*c*^	Targeted modification
Single-mode tools			
m6Anet (21)	Signal level	MIL-based neural network model	m6A
Nanom6A (22)	Signal level	XGBoost model	m6A
Tombo_de novo^*a*^ (24)	Signal level	Fisher’s method	Any
Comparative tools			
Differr (31)	Error rate	G-test	Any
DRUMMER (32)	Error rate	G-test and OR test	Any
ELIGOS2 (29)	Error rate	Fisher’s exact test	Any
EpiNano_Error^*b*^ (30)	Error rate	Z-score and linear regression	Any
Nanocompore (27)	Signal level	Two-GMM followed by logit test	Any
Tombo_com (24)	Signal level	KS-test (default), U-test, or *t*-test	Any
xPore (28)	Signal level	Multi-sample two-GMM followed by z-test	Any

^*a*^ Tombo provides three methods for RNA modification detection, including pre-computed model-based detection for specific modifications (only m5C available currently), model-free *de novo* method (Tombo_de novo), and detection through the comparison with an unmodified sample (Tombo_com). Two sample comparison models are provided, model_sample_compare and level_sample_compare. In the current study, the level_sample_compare model was applied.

^*b*^ The latest version of EpiNano (v1.2) can detect RNA modification using two distinct strategies, EpiNano-Error and EpiNano-SVM (30). EpiNano-Error was adopted in the present study to predict modified bases according to the differential base calling errors between two samples. EpiNano-SVM can be used with pre-trained models for m6A prediction or by building new models with features extracted from base-called data and raw signal data (9).

^*c*^ MIL, multiple instance learning; XGBoost, extreme gradient boosting; OR, odds ratio; GMM, Gaussian mixture model; KS, Kolmogorov-Smirnov.

Despite the wealth of existing methodologies detecting RNA modifications utilizing DRS data, caution should be exercised when considering which tool to use, especially when deciphering an unknown epitranscriptome without prior knowledge. To date, only a few studies utilized these computational methods to analyze vRNA modifications, most of which investigated human coronaviruses (9). Some of the studies used a combination of two methods, such as DRUMMER and Nanocompore (25), DRUMMER and ELIGOS2 (33), and Tombo_de novo and MINES (34). Tombo_com (26), Tombo_de novo (35), and m6Anet (36) were also applied individually. Although several detections were validated using orthogonal MeRIP-Seq, the authenticity of many predictions remains uncertain. There lacks a systemic evaluation for the performance of computational methods in mapping RNA modifications in the viral context.

Alphaviruses are a group of enveloped viruses containing a positive-strand genomic RNA (gRNA) of 11–12 kb. Most viruses in this genus are mosquito borne, and many can cause severe emerging and re-emerging infectious diseases (37, 38). Chikungunya virus (CHIKV) in this genus has brought several epidemic outbreaks throughout the tropical world and several subtropical areas since 2004 (39). A study showed that the CHIKV pre-replicated genomes are m6A modified within 2,000 nt at the 5′ end and the m6A-reader YTH-domain family (YTHDF) 1 binds and suppresses CHIKV replication (40). Attempts at investigating vRNA methylations have also been made with Sindbis virus (SINV), one of the most intensively investigated prototype alphaviruses. In the 1970s, the presence of m5C, m6A, and Nm decorations in intracellular SINV gRNAs and subgenomic RNA (sgRNA) was demonstrated by 3H labeling (41, 42). However, high-resolution position information on these modified nucleotides, which can be essential for studying their formation and function, is lacking. ONT DRS-based computational approaches can hopefully help locate different modifications, driving our understanding of alphavirus biology.

Here, we isolated SINV RNAs from infected mammalian and mosquito cells and synthesized the corresponding IVT RNAs, followed by ONT DRS. Obtained data were analyzed by 10 computational tools (Table 1) to detect potentially modified sites in the SINV RNAs and compare the tools’ performance. We found that single-mode methods failed to differentiate native and IVT samples and the cutoff values of certain comparative methods required optimization to decrease false-positive rates. Based on an integrated analysis of outputs from individual comparative methods, we proposed a pipeline combining Tombo_com and Xpore for vRNA modification detection and evaluated its performance using publicly available DRS and Me-RIP data of severe acute respiratory syndrome coronavirus 2 (SARS-CoV-2). Applying this pipeline, we then identified potentially modified locations in the SINV gRNAs and subgenomic RNAs (sgRNAs) produced in mammalian and mosquito cells, broadening the knowledge of alphavirus RNA modifications.

## RESULTS

### Pre-processing of ONT data

To obtain data sets for vRNA modification detection, we conducted ONT DRS for poly(A)+ RNAs isolated from SINV-infected baby hamster kidney BHK-21 and *Aedes albopictus* C6/36 cells. IVT RNAs were *in vitro* transcribed from a linearized plasmid DNA template containing the SINV infectious clone, the length of which was 11.7 kb, followed by polyadenylation and sequencing. The raw current signal data (FAST5 files) were base called using the Guppy workflow or the latest Dorado model.

**Fig 1:**
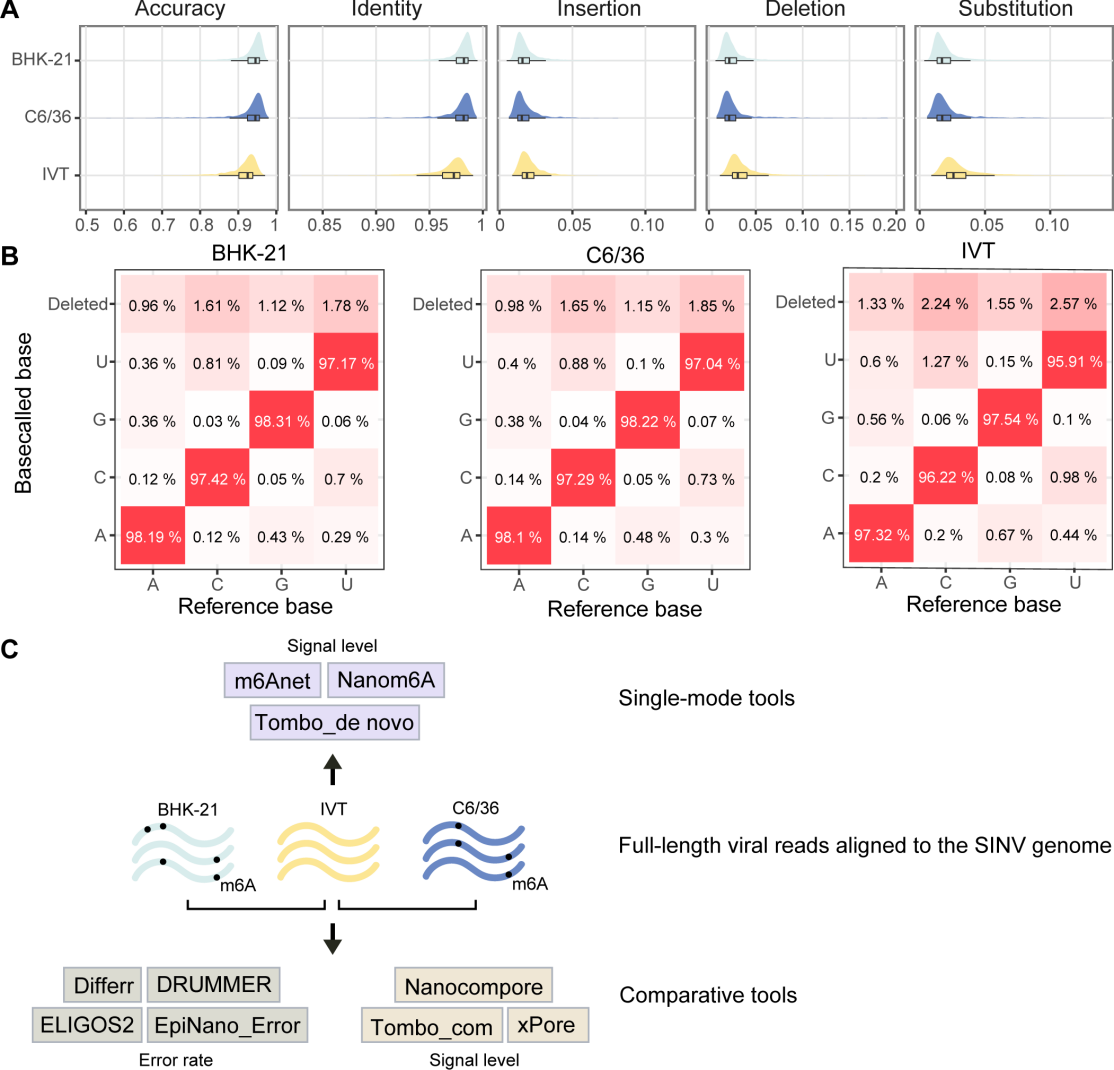
Data sets and computational tools used for mapping SINV RNA modifications. (**A**) Read-level data features of full-length viral reads base called using the Dorado model. Baby hamster kidney BHK-21 and *Aedes albopictus* C6/36 cells were infected with SINV at a multiplicity of infection of 0.1, followed by polyadenylated RNA isolation and ONT DRS. IVT RNA was synthesized from a plasmid encoding the SINV cDNA. Full-length viral reads were extracted from the alignment files. Read-level accuracy, identity, mismatch, insertion, and deletion were calculated using a custom Python script. (**B**) Base-level data features of full-length viral reads base called using the Dorado model. The confusion matrixes show the frequencies of each base being correctly base called, miscalled, or deleted in individual samples. (**C**) Classification of modification analysis tools used in the present study.

In the BHK-21 and C6/36 poly(A)+ RNA samples, a large number of viral reads were identified by alignment to the SINV genome using minimap2 (Table 2). To minimize potential biases resulting from sequencing coverage variance, full-length genomic reads, defined as those covering ≥90% of the reference genome, were subsampled from total viral reads. The BHK-21, C6/36, and IVT samples contained 5,600, 925, and 15,44 full-length reads, respectively. These reads showed even coverage depth along the viral genome (Fig. S1). Data features of full-length reads, including accuracy and errors at the level of reads and bases, were assessed based on the outputs from the Guppy (Fig. S2) or Dorado base caller (Fig. 1A and B). Essentially, Dorado yielded more accurate base calling results than Guppy. However, regardless of Dorado or Guppy base calling, the read accuracy of IVT was slightly lower than those of BHK-21 and C6/36 (Fig. 1A; Fig. S2A). IVT reads showed higher incidences of mismatch and indel than native reads. Regarding individual bases, the marginally poorer accuracies of IVT were observed across A, C, G, and U compared with BHK-21 or C6/36 (Fig. 1B; Fig. S2B). These results contradicted the generally accepted view that IVT RNA should be more accurately base called than wild-type RNA in DRS due to the lack of modifications. Nonetheless, similar results were reported in the context of SARS-CoV-2 (43). A parallel SINV IVT RNA sample was synthesized and sequenced, and the reads reproducibly showed slightly lower accuracy. The underlying reason remains to be discussed.

**TABLE 2:** Read statistics of ONT DRS reads aligned to the SINV genome

Parameter	BHK-21	C6/36	IVT^*a*^
Viral reads	608,790	222,344	26,493
Average length	2,708	2,484	3,588
Median length	2,674	2,112	2,575
N50 length	3,880	3,843	5,769
Full-length reads^*b*^	5,600	925	1,544
Subgenomic reads	133,554	66,857	N/A
Full-length read ratio (%)	0.92	0.42	5.83
Subgenomic read ratio (%)	21.94	30.07	N/A

^*a*^ IVT, *in vitro* transcribed.

^*b*^ Full-length reads were defined as those covering ≥90% of the reference genome.

Subsequently, full-length reads from different samples were subjected to RNA modification detection by different computational tools (Fig. 1C). A few approaches, such as m6Anet and Nanom6A, are designed to predict m6A decorations exclusively. The others are supposed to identify various types of RNA modifications, and all outputs were displayed and discussed in downstream analyses. Notably, for comparative methods, the error-based tools were developed utilizing the base calling mistakes of Guppy, so Guppy outputs were applied to these tools. On the other hand, the Dorado outputs were used for signal-based methods since the Dorado model generated more accurate base calling results, which allows the raw current signal to be more precisely assigned to each nucleotide.

### vRNA modification detection using 10 computational tools with default or recommended parameters

**Fig 2:**
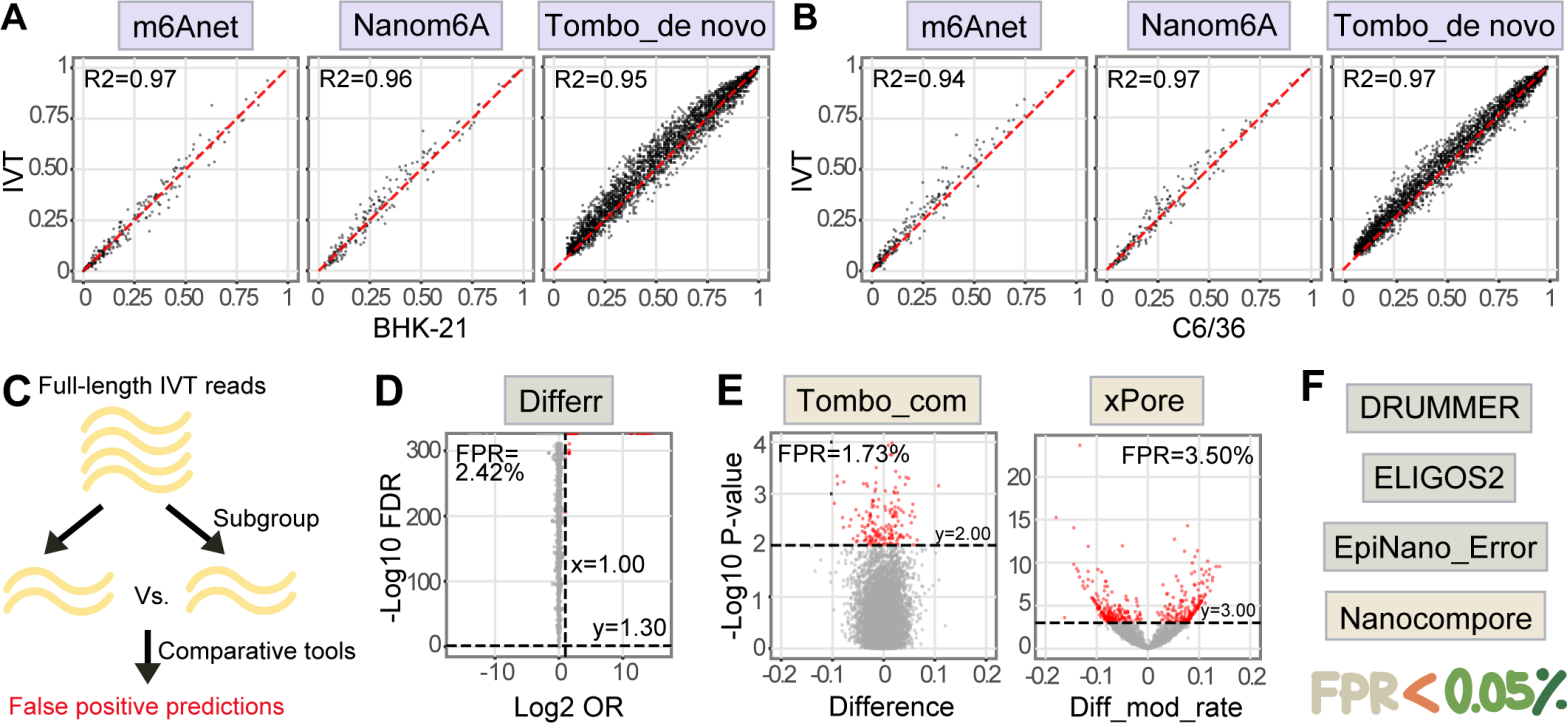
Evaluation of 10 computational tools in detecting vRNA modifications with default or recommended parameters. (**A**) Correlations between probability values computed for the IVT and BHK-21 samples at each testable site using single-mode methods. (**B**) Correlations between probability values computed for the IVT and C6/36 samples. For panels **A** and **B**, equal numbers of reads were subsampled from individual data sets, followed by analyses using m6Anet, Nanom6A, and Tombo_de novo; all predictable A sites are included. (**C**) Workflow of identifying false-positive predictions resulting from comparative approaches. Briefly, full-length IVT reads were divided into two subsets and used as input data sets for comparison method analyses. The default or recommended cutoffs were applied, and the final predictions were regarded as false positives. (**D**) Volcano-like plot for output from error rate-based comparative method Differr. (**E**) Volcano-like plots for outputs from current signal-based comparative methods Tombo_com and Xpore. For panels **D** and **E**, dashed lines indicate recommended cutoff values; false-positive predictions are labeled in red; all types of modifications are included. FPR, false-positive rate. (**F**) Comparative tools with a FPR lower than 0.05%.

The performance of the aforementioned 10 tools to detect vRNA modifications was evaluated. Three single-mode methods were assessed first using default parameters, including m6Anet, Nanom6A, and Tombo_de novo, which do not require comparison with a negative sample (Fig. 1C). The unmodified IVT reads were analyzed with the same procedures to exclude false-positive results. To eliminate possible biases caused by coverage sensitivity, 772 reads (half the amount of IVT reads) were randomly selected from each data set for analysis. Out of tested A sites, similar predictions were generated for different samples by individual methods (Fig. S3). The modification probability value calculated for the IVT sample at each testable motif was nearly identical to those calculated for the native BHK-21 or C6/36 samples (Fig. 2A and B), indicating that single-mode methods are inapplicable in the current settings.

Unlike single-mode approaches, comparative methods require or support the use of negative samples without or with low modifications. Four error-based methods (Differr, DRUMMER, ELIGOS2, and EpiNano_Error) and three approaches utilizing the current signal differences (Nanocompore, Tombo_com, and xPore) were tested in the current study (Fig. 1C). Given the high false-positive rate (FPR) of single-mode tools, the accuracy of comparative methods was first evaluated using the IVT sample. The 1,544 full-length IVT reads were stochastically divided into two groups, which were used as the input data set pair for comparison (Fig. 2C). Ideally, as the two subgroups originated from the same unmodified sample, none of the nucleotides were supposed to be recognized as modified. However, when applying the recommended thresholds (Table 3), Differr, Tombo_com, and xPore generated hundreds of modification predictions (Fig. 2D and E; Fig. S4). The false-positive identification process was repeated 100 times, and the average FPR calculated for Differr, Tombo_com, and xPore was 2.42%, 1.73%, and 3.50%, respectively. The FPRs of DRUMMER, ELIGOS2, EpiNano_Error, and Nanocompore were lower than 0.05% (Fig. 2F). To mitigate frequent false-positive predictions, the outputs of Differr, Tombo_com, and xPore can be improved by tuning cutoff values, including significance (false discovery rate or *P*-value) and effect size (odds ratio, difference, or differential modification rate).

**TABLE 3:** Recommended and optimized cutoffs for comparative tools used in the present study

Method	Statistic^*a*^	Recommended cutoff	Optimized cutoff	Cutoff for analyses
Differr (31)	−Log10 FDR	1.30	N/A	1.30
	Log2 OR	1	2.34	2.34
DRUMMER (32)	−Log10 adj-*P*	1.30	N/A	1.30
	Log2 OR	0.58	0.93	0.93
ELIGOS2 (29)	−Log10 *P*-value	3	2	3
	Log2 OR	0.26	0.83	0.83
EpiNano_Error (30)	Built-in parameters	N/A	N/A	N/A
Nanocompore (27)	−Log10 *P*-value	2	2	2
	|Log2 OR|	0.50	0.44	0.50
Tombo_com (24)	−Log10 *P*-value	2	2	2
	|Difference|	N/A	0.05	0.05
xPore (28)	−Log10 *P*-value	3	7	7
	|Diff_mod_rate|	N/A	0.09	0.09

^*a*^ FDR, false discovery rate; OR, odds ratio; adj-*P*, adjusted *P*-value; Diff_mod_rate, differential modification rate.

### Cutoff optimization for comparative methods

**Fig 3:**
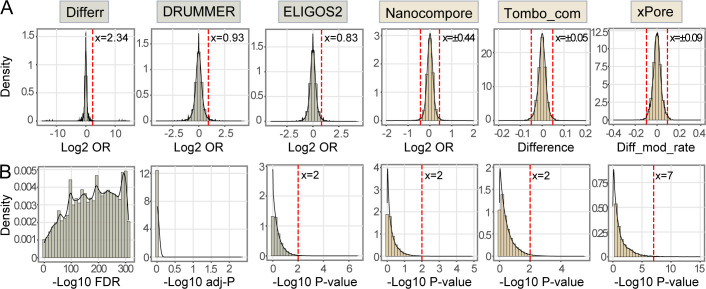
Cutoff optimization for comparative methods based on unmodified IVT reads. (A) Effect size cutoff values determined by computing 99% confidence intervals (CIs). One-sided 99% CIs were calculated for error-based methods, and two-sided 99% CIs were computed for signal-based approaches. (B) Significance cutoff values decided by computing percentile. The final percentiles were rounded to the nearest integers and used as new significance thresholds.

The cutoffs of a few comparative methods (Differr, Tombo_com, and xPore) need to be adjusted to exclude false-positive results (Fig. 2D and E). Although DRUMMER, ELIGOS2, and Nanocompore produce low rates of false-positive predictions using the recommended parameters, their cutoff values were also examined to obtain the optimized ones. EpiNano_Error uses built-in thresholds and outputs significant predictions directly, thereby not being inspected here. To this end, full-length genomic IVT reads were randomly partitioned into two subsets, the coverage depths of each set being 772, followed by analyses using comparative methods. Significance and effect size values were generated for each site of the reference genome (*n* = 11,703). This procedure was repeated 100 times. Resultant significance and effect size values were pooled separately (*n* = 1,170,300). Each tool’s effect size values or corresponding logarithmic values roughly followed a normal distribution (Fig. 3A). One-sided 99% confidence intervals (CIs) were calculated for error-based methods, and two-sided 99% CIs were computed for signal-based approaches (Fig. 3A). In most cases, the resulting new effect size cutoffs were more stringent than the original ones, except for Nanocompore (Table 3). Nanocompore, therefore, adopted the originally recommended effect size threshold for subsequent analyses. Regarding significance, each method’s negative logarithms of significance values were ordered from smallest to largest (Fig. 3B). The final percentiles were rounded to the nearest integers and used as new significance cutoffs for ELIGOS2, Nanocompore, Tombo, and xPore (Table 3). The distribution of Differr significance values was turbulent. Meanwhile, DRUMMER displayed extremely concentrated significance value distributions. Therefore, assigning new significance thresholds to these two methods was difficult. Their recommended significance cutoffs were used afterward.

Cutoff optimization was also conducted under different coverage depths, ranging from 20 to 772. It was shown that higher coverage was favorable for detection sensitivity (Table 4). In all cases, the effect size cutoff values decreased as coverage rose. The significance thresholds for Tombo_com were stable across different sequencing coverages. For xPore, the optimized significance cutoff values (−log10 *P*-value) changed from 7 (at coverage 772) to 14 (at coverage 20). These data suggested that the cutoff values need to be adjusted at different coverage depths to reduce false-positive predictions while maintaining high detection sensitivity.

**TABLE 4:** Optimized cutoffs at different sequencing coverage depths

Method	Statistic	772×	500×	250×	100×	50×	20×
Differr	−Log10 FDR	N/A	N/A	N/A	N/A	N/A	N/A
	Log2 OR	2.34	3.31	5.87	10.87	16	24.30
DRUMMER	−Log10 adj-*P*	N/A	N/A	N/A	N/A	N/A	N/A
	Log2 OR	0.93	1.11	1.48	2.02	2.33	2.60
ELIGOS2	−Log10 *P*-value	2	2	2	2	2	1
	Log2 OR	0.83	1.01	1.38	1.92	2.29	2.30
Nanocompore	−Log10 *P*-value	2	1	N/A	N/A	N/A	N/A
	|Log2 OR|	0.40	0.52	0.74	1.07	1.55	1.90
Tombo_com	−Log10 *P*-value	2	2	2	2	2	2
	|Difference|	0.05	0.06	0.08	0.13	0.19	0.30
Xpore	−Log10 *P*-value	7	7	8	8	10	14
	|Diff_mod_rate|	0.09	0.1	0.14	0.20	0.30	0.50

### Integrated analysis of multiple comparative methods

The above-described comparative methods were applied to make comparisons between the native and IVT viral reads to identify potential modification sites in the SINV genome. An equal number of reads, 772 in amount, were randomly subsampled from the BHK-21, C6/36, and IVT full-length viral reads. The adjusted cutoff values were applied accordingly (Table 3). The outputs from different computational tools varied, and each method resulted in distinguishable predictions for vRNAs produced in BHK-21 and C6/36 cells (Fig. S5).

**Fig 4:**
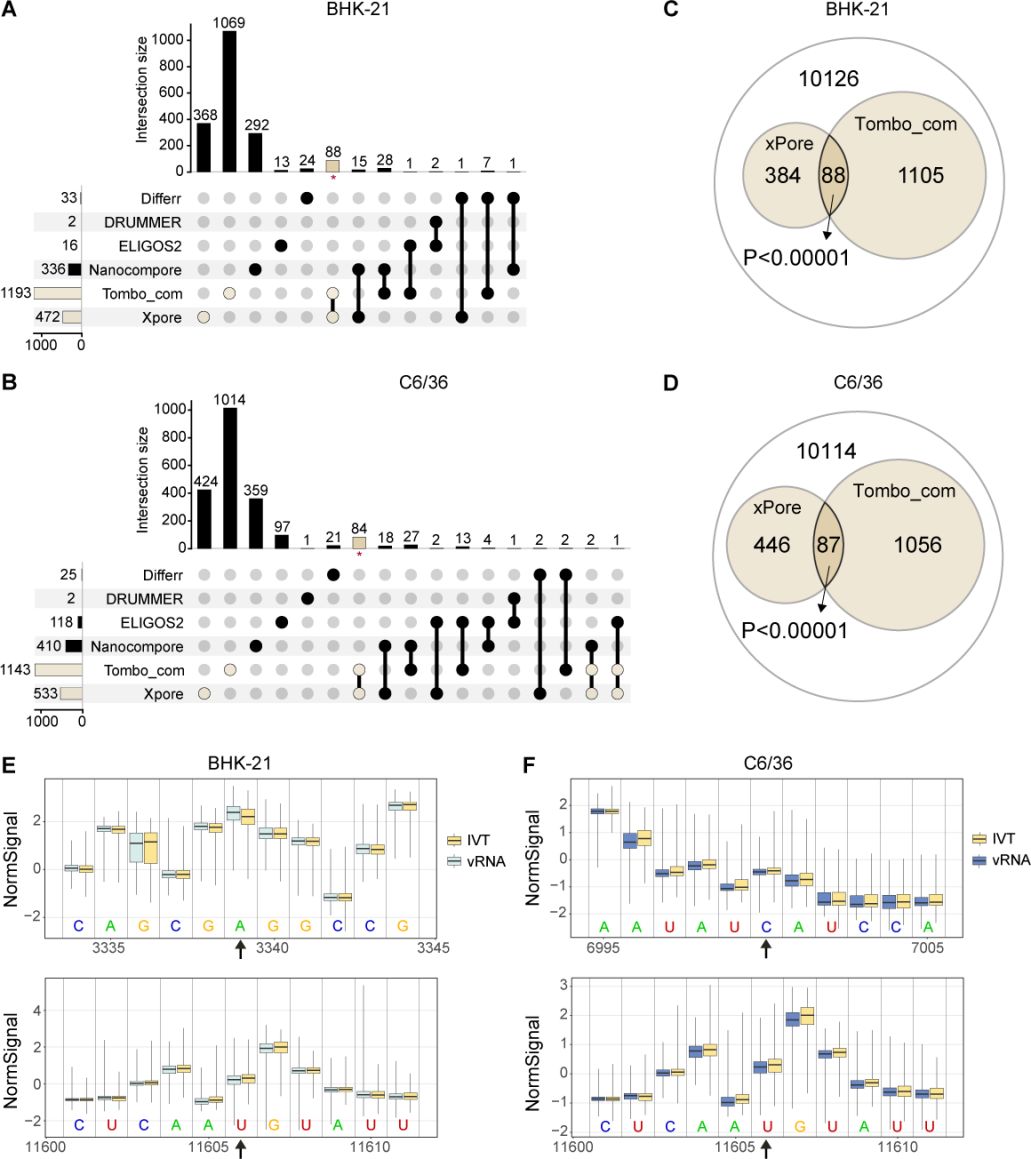
Integrated analysis of comparative methods. An equal number of reads, 772 in amount, were randomly subsampled from the BHK-21, C6/36, and IVT samples, followed by analyses using comparative methods. (A and B) UpSet plots for modification sites reported by different comparative methods in the BHK-21 or C6/36 samples. Red asterisks indicate significantly enriched intersections from the background. (C and D) Veen diagrams showing the intersections of Tombo_com and xPore predictions. The *P*-values were calculated by Fisher’s exact test. (E and F) Boxplots showing normalized signal differences at predicted modification sites and adjacent regions. The plots were generated using Tombo. Predicted sites are indicated by black arrows.

It is suggested that integrating results from multiple tools can greatly improve performance (44). Therefore, the modification sites reported by individual comparative tools were pooled to identify intersected predictions (Fig. 4A and B). Out of the total predictions, only a limited number of sites were recognized by at least two approaches. Fisher’s exact test was applied to measure whether individual intersections were significantly enriched from the background. The results showed that only the Tombo_com and xPore intersections yielded *P*-values below 0.001 in either BHK-21 or C6/36 samples (Fig. 4C and D). In this way, 88 and 87 potentially modified positions were detected in the BHK-21 and C6/36 SINV genomes, respectively. As both Tombo_com and xPore are current signal-based methods, the differences in normalized signals (Fig. 4E and F) as well as in corresponding raw signal traces (Fig. S6) at the predicted modification sites were checked. It was further determined if the combo of Tombo_com and xPore were applicable at different coverage depths. Native and IVT reads were subsampled to certain equivalences, followed by analyses using Tombo_com and xPore with the corresponding optimized cutoff values. The intersection of Tombo_com and xPore at each coverage depth was tested by Fisher’s exact test. The enrichment was not significant (*P* < 0.001) until coverage reached 500 (Fig. S7). At the coverage of 500, 77 and 72 sites were recognized in the BHK-21 and C6/36 samples, respectively.

**Fig 5:**
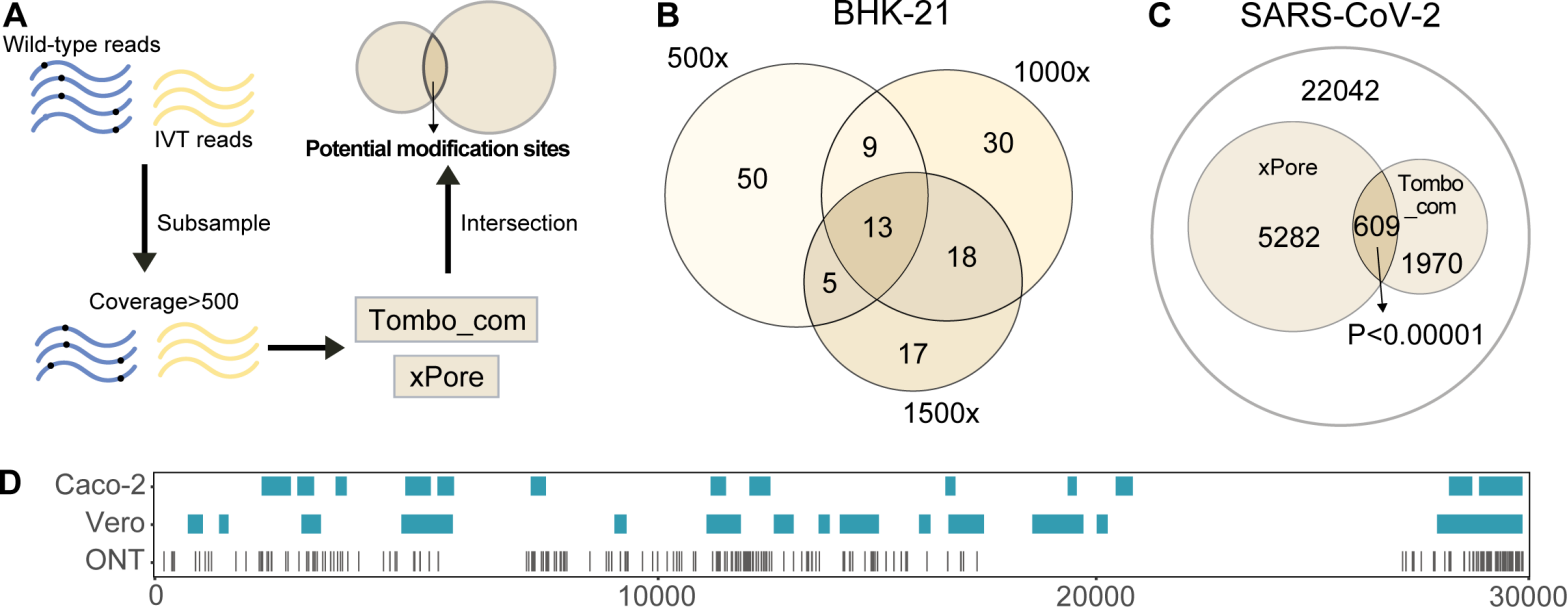
Proposal of a vRNA modification detection pipeline and its performance evaluation. (A) Proposed pipeline for detecting vRNA modifications utilizing computational methods. Briefly, native and IVT reads are subsampled to an equal coverage (>500), followed by Tombo_com and xPore analyses and intersection identification. Cutoff values at the coverage depth of 500 are recommended for use. (B) Venn diagram showing the relationship between modification sites detected at different coverage depths while using the same cutoff values. Different numbers of reads (500, 1,000, and 1,500) were randomly subsampled from the IVT and BHK-21 full-length viral reads and analyzed the Tombo_com and xPore pipeline. (C) Detection of modification sites in the SARS-CoV-2 vRNAs using the Tombo_com and xPore pipeline. DRS fats5 data were obtained from reference 26. (D) Comparison between the computational pipeline outputs and MeRIP data in detecting SARS-CoV-2 vRNA m6A modifications. Modified A sites were extracted from the total prediction result of the Tombo_com and xPore pipeline. MeRIP data were obtained from reference 45. Caco-2 and Vero indicate MeRIP data measured in Caco-2 and Vero cells, respectively.

Based on these data, we proposed a pipeline for detecting potential vRNA modifications using computational tools (Fig. 5A). Briefly, native RNA and IVT samples are prepared and sequenced using the ONT DRS platform. Subsequently, an approximately equal number of reads, the minimum coverage depth of which is 500, are subsampled from the two data sets. Following Tombo_com and xPore analyses, their intersections are the potential modification sites. It is notable that the location of a real modification can be either at or near the reported site, as the position impacted to the greatest extent is dependent on the sequence context (45). Using a pair of dedicatedly prepared samples, such as m6A-containing and m6A-deficient RNAs, can facilitate the localization of real modifications. Moreover, it is supposed that the optimized cutoffs at different coverage depths should be applied accordingly (Table 4). However, this requirement is somewhat redundant since there were trivial cutoff differences at coverage 500 and 772 for Tombo_com and xPore (Table 4). Moreover, higher coverage generated fewer predictions given a threshold (Fig. S8). Comparison between the resulting modification sites revealed that at the same cutoffs, higher coverage favored detection, as 67.9%, 57.1%, and 35.1% of sites were repeatedly predicted at coverage depths of 1,500, 1,000, and 500, respectively (Fig. 5B). Therefore, regardless of sequencing depth, the cutoffs at coverage 500 are recommended to filter Tombo_com and xPore results to simplify the pipeline. The script for this pipeline is available at https://github.com/lrslab/Scripts_merge_DRS_methods.

To evaluate its performance, the Tombo_com and xPore pipeline was applied to publicly available DRS FAST5 data of SARS-CoV-2 (26). Native viral reads were generated from total RNAs extracted from Vero cells infected with SARS-CoV-2 (BetaCoV/Korea/KCDC03/2020). IVT RNAs were *in vitro* transcribed from viral gRNA reverse transcription-PCR products using T7 RNA polymerase. The reads were downsampled to balance the coverages between native and IVT samples (approximately 5,000), followed by the Tombo_com and xPore pipeline analyses. The intersections consisted of 609 modification sites (Fig. 5C), including 190 A sites. Compared with the MeRIP data (46), which showed the m6A positions in the SARS-CoV-2 (USA-WA1/2020) vRNAs produced in the Vero and Caco-2 cells, 104 predicted A sites (54.7% of the total predicted A sites) were located within the m6A peaks (Fig. 5D). As many more 2′-O-methyladenosines (Am) exist in the SARS-CoV-2 vRNAs compared with m6A (46), the majority of predicted A sites can be Am modifications.

### Potential modification sites in SINV RNAs

**Fig 6:**
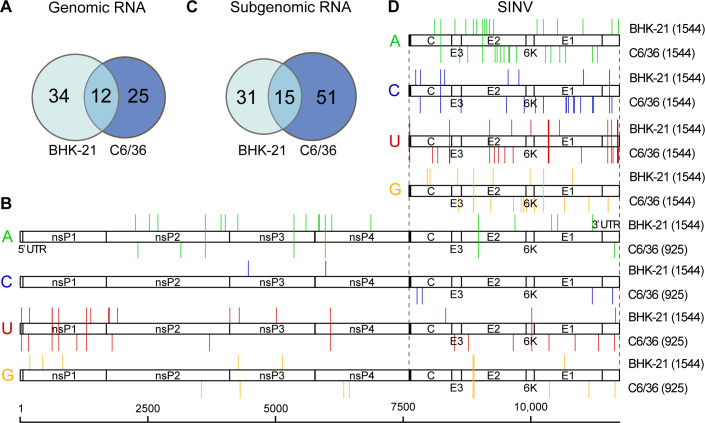
Predictions of modification sites in the gRNAs and sgRNAs. As many reads as possible were subsampled from the original sequencing data while maintaining balanced coverage depths for the input dataset pairs, followed by analysis using the Tombo_com and xPore pipeline. (A) Intersections of modification sites in the SINV gRNAs generated in BHK-21 and C6/36 cells. (B) Positions of predicted modification sites in the SINV gRNAs. Read coverage depths of individual analyses are indicated in parentheses. (C) Intersections of modification sites in the SINV sgRNAs, generated in BHK-21 and C6/36 cells. (D) Positions of modification sites in the SINV sgRNAs.

The data sets were re-analyzed using the Tombo_com and xPore pipeline to identify potential modification sites in the SINV gRNA and sgRNA. As many reads as possible were used for analysis while maintaining balanced coverage depths for the input data set pairs. A total number of 5,600, 925, and 1,544 full-length reads were obtained from the BHK-21, C6/36, and IVT sequencing data, respectively (Table 2). Therefore, a coverage depth of 1,544 was applied for the comparison between BHK-21 and IVT; a coverage depth of 925 was adopted when comparing C6/36 with IVT. Cutoff values obtained at coverage 500 were used to filter the prediction outputs. As a result, 46 and 37 potential modification sites were identified in the SINV gRNAs produced in BHK-21 and C6/36 cells, respectively (Table 5; Tables S1 and S2), 12 of which were shared (Fig. 6A). Their positions are illustrated in Fig. 6B. Notably, six modified U sites were presented in both BHK-21 and C6/36 SINV gRNAs, with four of them clustered at the 5′ end of the gRNA. In addition, four modified A sites were commonly recognized and dispersed within the gRNA body.

**TABLE 5:** Predicted modification sites in the SINV gRNA and sgRNA

		BHK-21	C6/36	Intersection
gRNA	A	19	7	4
	U	17	17	6
	C	2	4	0
	G	8	9	2
	Total	46	37	12
sgRNA	A	17	17	2
	U	13	19	9
	C	8	17	2
	G	8	13	2
	Total	46	66	15

sgRNA reads were also extracted from the original sequencing data (Table 2). Potential sgRNA modifications were detected. A coverage depth of 1,544 was applied. With the same analysis matrix, 20 more modification sites were detected in sgRNAs produced in C6/36 cells than in BHK-21 cells (Table 5; Tables S3 and S4). Fifteen modification sites were shared by sgRNAs derived from the two cell lines (Fig. 6C), most of which were modified uridines. It is seen that four modified uridines were centered in the 3′ untranslated region (UTR), while the remaining were scattered along the structural genes (Fig. 6D). Compared with the same region in the gRNA, sgRNAs showed more intensive modifications (Fig. 6B and D).

## DISCUSSION

The advent of ONT DRS provides a new strategy to identify modified ribonucleotides in RNA molecules. To this end, dozens of computational tools are developed by exploiting the differences at the signal level or the increased base calling error rates caused by modifications. These tools were designed to map eukaryotic epitranscriptomes and were tested on specific species, such as humans, mice, plants, and yeasts. However, little is known about their capabilities and limitations. A recent publication systematically compared 10 of these computational tools for mapping m6A in human and mouse transcriptomes (44). It revealed a trade-off between precision and recall. Multiple factors, such as motif preference, sequencing depth, and m6A stoichiometry, can potentially affect the prediction results. A few computational tools have been used to analyze vRNA modifications, mostly coronavirus epitranscriptomes (9). Given that viruses exploit the host machinery to complete their life cycle, it is plausible that they also co-opt host enzymes to modify nascent vRNAs. However, the detail remains elusive. In most cases, the vRNA modification profile should be regarded as an unknown epitranscriptome without prior knowledge. Therefore, extra caution needs to be exercised when using RNA modification mapping computational tools.

In the present study, we assessed the performance of 10 computational tools in predicting modification sites in vRNAs. Native vRNAs, including genomic and subgenomic RNAs, were obtained from two cell lines infected with the prototype alphavirus SINV. IVT RNA was synthesized from a plasmid containing the SINV cDNA. We found that all the tested single-mode methods (m6Anet, Nanom6A, and Tombo_de novo) showed low validity, as the modification probability values given for native and IVT RNAs were almost indistinguishable (Fig. 2A; Fig. S3). With recommended thresholds, three comparative tools (Differr, Tombo_com, and xPore) also generated many false-positive predictions for the IVT reads (Fig. 2B; Fig. S4). Therefore, we adjusted the cutoff values of different comparative tools and found that a higher coverage was favorable for detection sensitivity (Fig. 3; Table 4). Native and IVT RNAs were subsequently analyzed using seven comparative methods (Differr, DRUMMER, ELIGOS2, EpiNano_Error, Nanocompore, Tombo_com, and xPore) using optimized cutoff values at the coverage of 772 (Fig. S5). An integrated analysis of the outputs from individual tools suggested that the intersected predictions of Tombo_com and xPore were significantly enriched from the background (Fig. 4). According to these findings, we proposed a pipeline for vRNA modification detection using computational tools (Fig. 5A). Shortly, equal amounts of native and IVT DRS reads with a minimum coverage of 500 are subsampled from total data sets, followed by Tombo_com and xPore analyses. The optimized cutoffs at the coverage depth of 500 are recommended for use. Moreover, as many reads as possible should be included in the analyses since higher coverage depth helps decrease low-confidence predictions (Fig. 5B). The intersections of Tombo_com and xPore outputs are predicted modification sites. We subsequently applied this pipeline to publicly available SARS-CoV-2 DRS data to evaluate its performance. The final predictions included 190 A sites, 54.7% of which were located within the MeRIP peaks (Fig. 5C and D).

In our pipeline, the two tools used, Tombo_com and xPore, are comparative approaches that utilize the current signal differences. Methods relying on base calling errors led to varied predictions (Fig. 4A and B; Fig. S5). DRUMMER and EpiNano_Error gave very few or no outputs based on our native and IVT data sets, showing false-negative predictions. On the other hand, Differr and ELIGOS2 suggested dozens of modification sites. This difference may result from the statistical methods used by individual tools. DRUMMER and EpiNano_Error run two statistical tests to identify modified bases, thereby outputting very few predictions. Moreover, tools utilizing base calling errors overlook positions where mismatches and indels occur in the IVT sample, even though they are expected to represent a very small portion. However, a nonnegligible number of mismatches and indels were observed in our IVT reads, leading to lower accuracies compared with the native reads regardless of base callers used (Fig. 1A and B; Fig. S1). An IVT replicate was prepared and sequenced, the result of which consolidated this issue. The corresponding FAST5 data were uploaded to the Figshare repository together with other data sets. Additionally, a lower accuracy of IVT reads was also reported in the context of SARS-CoV-2 (43). In our data, IVT reads showed lower accuracies across A, C, U, and G without evident biases (Fig. 1B). We also examined the error rates within and outside homopolymer regions (defined as at least four consecutive identical bases), demonstrating that the poorer accuracies of IVT reads existed across the entire genome rather than being restricted to the homopolymer sequences. Despite these findings, the reason remains to be elucidated. Nevertheless, as transcriptional errors introduced by SP6 RNA polymerase have been reported (47, 48), it is possible that some transcriptional errors were introduced during *in vitro* synthesis considering the long length of synthesized RNA molecules (approx. 11.7 k). This hypothesis can be tested via short-read sequencing in the future. In contrast to error-based methods, signal-based methods directly compare the current signals without filtering errors in IVT reads, generating more predictions (Fig. 4A and B; Fig. S5).

Using the Tombo_com and xPore pipeline, we identified 46 and 37 potential modification sites in BHK-21 and C6/36 SINV gRNAs and 46 and 66 modification sites in sgRNAs. Among these sites, modified uridines were recognized frequently and showed a good overlap between SINV RNAs generated in BHK-21 and C6/36 cells (Fig. 6B and D). This can hint at uridine modifications, most likely pseudouridines, in SINV gRNA and sgRNA molecules, but orthogonal measurement is required for validation. On the other hand, the interpretation of other prediction results is ambiguous. A previous study demonstrated the presence of m5C, m6A, and Nm in intracellular SINV gRNAs produced in BHK and chick embryo fibroblast cells, with m5C accounting for the majority (41). However, using the Tombo_com and xPore pipeline, very few modified C sites were identified and showed no overlapping in the intracellular gRNAs generated in BHK-21 and C6/36 cells. Instead, many modified A and G sites were reported. There can be several explanations. Most importantly, the truly modified nucleotides can be near the reported ones, as modifications can have impact on the current signal and dwell times of adjacent sequences and also distal positions (approximately 10 nt in the 3′ direction) through interactions with the helicase motor protein (49). Second, we collected cellular total RNA at 24 h post-infection when extracellular viruses reached the highest titer. In other words, intracellular gRNAs at this time point were mostly used for assembly and resembled virion gRNAs. As extracellular virion gRNAs were shown to have limited internal methyl modifications (41), it is plausible that intracellular gRNAs at this time point also contained few methylated nucleotides internally. Finally, the predicted modifications may be other than methylation, such as A to I editing (11). Our pipeline cannot differentiate these specific modification types.

In all, in the present study, we compared the performance of different computational tools and proposed a pipeline integrating Tombo_com and xPore to predict potential modification sites in vRNAs based on ONT DRS data. Despite a supportable validation using publicly available SARS-CoV-2 data, we are cautious about the use of the Tombo_com and xPore pipeline. The prediction results greatly changed when we subsampled different amounts of reads from the data sets or applied different cutoffs. We recommend high coverage depths and stringent cutoffs for analysis to reduce false positives. It is also notable that segmentation errors of raw signals cannot be completely ruled out in our analysis, which may cause false identification of modification sites. Therefore, caution should be taken when interpreting the prediction results, and careful orthogonal experimental validation is warranted. Nonetheless, computational tools provide an alternative strategy to identify RNA modifications. As the new ONT DRS kit (SQK-RNA004) is around the corner, we can expect substantial progress in the detection validity and reliability of computational tools. Additionally, we neglected stoichiometry information on individual modification sites here. Comparative methods usually fail to support single-molecule modification analysis and prevent stoichiometry prediction despite xPore being claimed to calculate the proportions of modified nucleotides at single positions (28). Improvements in stoichiometry analysis will allow more detailed profiling of RNA modifications in the future.

## MATERIALS AND METHODS

### Virus infection and RNA isolation

The full-length cDNA clone of SINV strain Toto 1101 was kindly provided by Charles Rice’s lab at Rockefeller University, USA. The virus was rescued as previously described (50). *Aedes albopictus* C6/36 cell and baby hamster kidney BHK-21 cell were purchased from the American Type Culture Collection, Manassas, VA, USA. Cells were maintained as previously described (50).

Cells were seeded into six-well plates at a density of 5 × 10^5^ cells/well and inoculated with SINV at a multiplicity of infection of 0.1. After 1 hour of incubation, the cell monolayers were washed three times with phosphate-buffered saline and covered with 1-mL cell culture medium. The supernatant was removed post-infection at the designated time point, and 800 μL TRIzol Reagent (Invitrogen, Waltham, MA, USA) was added to the cell monolayer. Phase separation and total RNA precipitation were performed according to the manufacturer’s instructions. The RNAs of SINV-infected C6/36 cells were isolated at 3 days post-infection (dpi). The RNAs of SINV-infected BHK-21 cells were isolated at 1 dpi.

### *In vitro* transcription

The plasmids containing the SINV infectious clone were purified by the Plasmid Maxi Kit (Qiagen, Hilden, Germany), linearized by digestion with NotI (New England Biolabs, Ipswich, MA, USA), and purified by phenol-chloroform extraction. RNAs were then synthesized in an *in vitro* transcription system by incubating at 37°C for 1 h containing the linearized DNA template, NTP mix, dithiothreitol, RNaseOUT ribonuclease inhibitor, and SP6 RNA polymerase (Thermo Fisher Scientific, Waltham, MA, USA). Following DNase I treatment, LiCl Precipitation Solution (Invitrogen) was added to purify the synthesized RNAs.

### ONT DRS and data processing

Poly(A)+ RNAs were enriched from total RNAs using Dynabeads mRNA Purification Kit (Invitrogen). IVT RNAs were polyadenylated using *Escherichia coli* poly(A) polymerase (New England Biolabs). A total of 1,000 ng of poly(A)+ RNAs were subjected to DRS library preparation using an SQK-RNA002 kit (Oxford Nanopore Technologies, Oxford, UK). The optional reverse transcription step was included using SuperScript III Reverse Transcriptase (Invitrogen). Sequencing was performed on the MinION platform using R9.4.1 flow cells (Oxford Nanopore Technologies).

Reads were base called using the Guppy workflow (v5.0.16) with the configuration of rna_R9.4.1_70bps_hac.cfg or using the Dorado workflow (v0.3.4) with the configuration of rna002_70bps_hac@v3. The resulting FASTQ files were aligned to the SINV genome (GenBank: NC_001547.1) using Minimap2 v2.17 with parameter settings “-ax map-ont” (51). Mapping results were stored in SAM files, which were subsequently converted into bam files and sorted and indexed using SAMtools v1.17 (52). Read alignment information was extracted using a custom Python script based on Pysam. Full-length viral reads, defined as those covering ≥90% of reference lengths, and subgenomic reads were subsampled from total viral reads with a custom script.

### Detection of modification sites using diverse computational tools

Ten computational tools were run with parameter settings described as follows.

m6Anet (v1.1.1) relies on the eventalign module in Nanopolish (v0.14.0) to assign raw current signals to each nucleotide. The signal features, including mean, standard deviation, and signal length, are used as input to a Multiple Instance Learning-based neural network model. Probability-modified values are generated for each DRACH k-mer. An empirical threshold was set as a probability value greater than 0.9.

Nanom6A (v2.0) utilizes the Tombo resquiggling process to assign the current signals to each nucleotide. It extracts features from normalized current signals for each read, including the median, mean, standard deviation, and dwell time. These features are used as input for an Extreme Gradient Boosting machine learning model, and a modification probability is generated for each RRACH k-mer. Sites with a modification probability greater than 0.5 were classified as modified.

Tombo_de novo (v1.5.1) performs the resquiggling process on all raw reads, followed by a hypothesis test statistically comparing the current signal with the canonical model based on the genome sequence for each read at each position. An empirical threshold was set as a probability value greater than 0.9 (53).

Differr (v0.2) utilizes alignment files for the sample of interest and a standard negative control as input. The prediction of modified bases was determined by the false discovery rate and odds ratio (OR) obtained from the statistical test results.

DRUMMER (v1.0) uses two alignment files as input. Putative modification sites are defined as those with both adjusted *P*-values from the *G*-test and OR test less than a specified input and the OR test result exceeding a specified value. The present study employed the OR test adjusted *P*-value and the OR test result to simplify graphic visualization and data analysis.

ELIGOS2 (v2.1.0) detects RNA modifications by comparing two alignment files. The present study relaxed the threshold values for the *P*-value and OR to obtain all outputs from the statistical test, followed by custom filtering using specified cutoffs.

EpiNano_Error (v1.2) utilizes the differences between two alignment files in base calling errors (mismatches, insertions, and deletions) and the alterations in per-base qualities to predict modified bases. The present study applied the combination of all base calling errors for prediction (-f sum_err). The output took the default threshold.

Nanocompore (v1.0.4) collapses the outputs from the Nanopolish eventalign module to generate files containing the median intensity and dwell time, followed by pairwise comparisons of the collapsed results from the sample of interest and a control sample. The prediction of modified sites was determined by *P*-value and OR from the Gaussian mixture model logit test.

Tombo_com (v1.5.1) is the canonical sample comparison method provided by Tombo. It offers two distinct ways for modified base detection “model_sample_compare” and “level_sample_compare.” The latter approach was adopted in the present study. The “--store-p-value” option was applied to save *P*-values for subsequent analysis. The “text_output” option was employed to extract *P*-value and difference statistics.

xPore (v2.1) employs the outputs derived from the Nanopolish eventalign module for all samples to generate a configuration file. The prediction of modified positions was determined by *P*-value and differential modification rate from the statistical test results.

### SARS-CoV-2 m6A modification validation

ONT DRS FAST5 data for SARS-CoV-2 were from reference (26). The corresponding FASTQ files were aligned to the SARS-CoV-2 genome (GenBank: NC_045512.2) using minimap2 with options “-ax map-ont.” Viral reads were subsampled to a coverage depth of around 5,000 across the whole genome. Tombo_com and xPore were applied to generate initial predictions. xPore failed to process the data sets originally, which is likely due to the fragmented nature of IVT RNAs. To address this problem, a customized reference genome was created by dividing the SARS-CoV-2 genome sequence into several segments (about 1,500 nt each) to correspond to individual IVT fragments. In this way, the Tombo_com and xPore pipeline was successfully applied to SARS-CoV-2. The final output was examined by intersecting it with published MeRIP data (46), which showed m6A peaks in the SARS-CoV-2 vRNAs produced in Vero and Caco-2 cells. By taking the union of peaks called in these two cell types, the ratio of predicted modified A sites within the m6A peaks was calculated against total modified A sites.

## Data Availability

All FAST5 files used for vRNA modification detection in the current study are deposited at https://figshare.com/projects/SINV_vRNA_modification_detection_by_Nanopore_DRS/167354. Corresponding FASTQ files are available in the NCBI BioProject database under accession number PRJNA983926. All code scripts related to this article are deposited at https://github.com/lrslab/DRS_vRNA_modification_content.

## SUPPLEMENTAL MATERIAL

Supplemental figures msystems.01163-23-s0001.pdf Fig. S1 to S8.

Supplemental tables msystems.01163-23-s0002.xlsx Tables S1-S4

ASM does not own the copyrights to Supplemental Material that may be linked to, or accessed through, an article. The authors have granted ASM a non-exclusive, world-wide license to publish the Supplemental Material files. Please contact the corresponding author directly for reuse.
