# Supplementary material for: Utilization of nanopore direct RNA sequencing to analyze viral RNA modifications
Source: mSystems. 2024 Jan 31;9(2):e01163-23. doi: 10.1128/msystems.01163-23 (PMC10878088; doi:10.1128/msystems.01163-23)
Supplement: Supplemental figures — Fig. S1 to S8. [file msystems.01163-23-s0001.pdf]

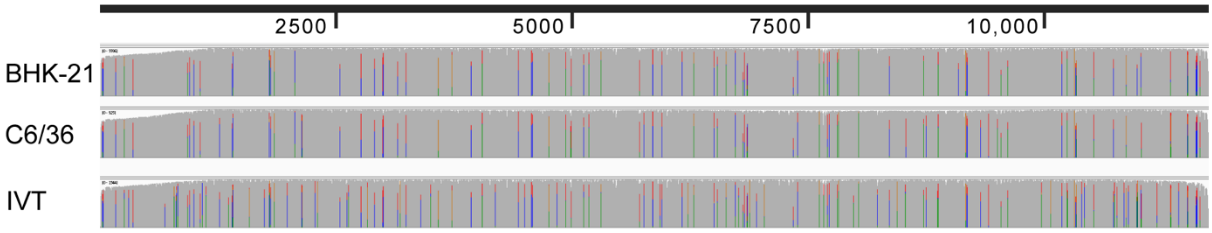

Figure S1 Integrative Genomics Viewer (IGV) image of full-length viral reads aligned to the Sindbis virus (SINV) genome.

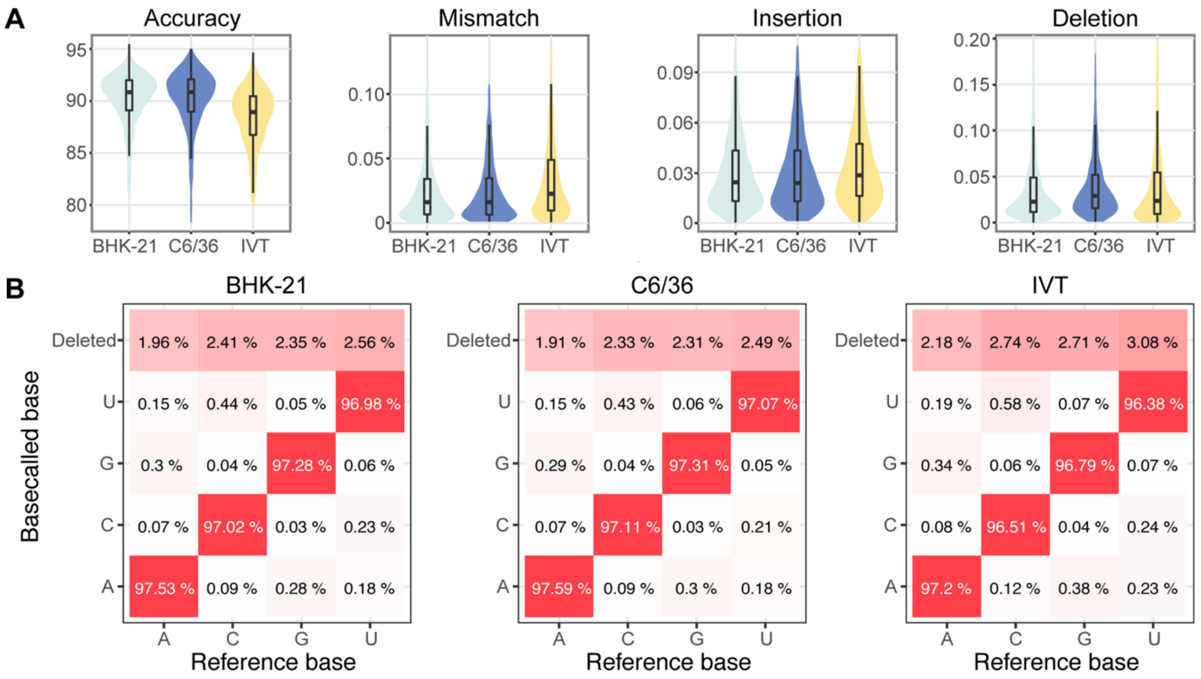

Figure S2 Data features of full-length viral reads basecalled using the Guppy basecaller. (A) Read-level data features. Read-level accuracy, mismatch, insertion, and deletion were calculated using a custom Python script. (B) Base-level data features. The confusion matrixes show the frequencies of each base being correctly basecalled, miscalled, or deleted in individual samples.

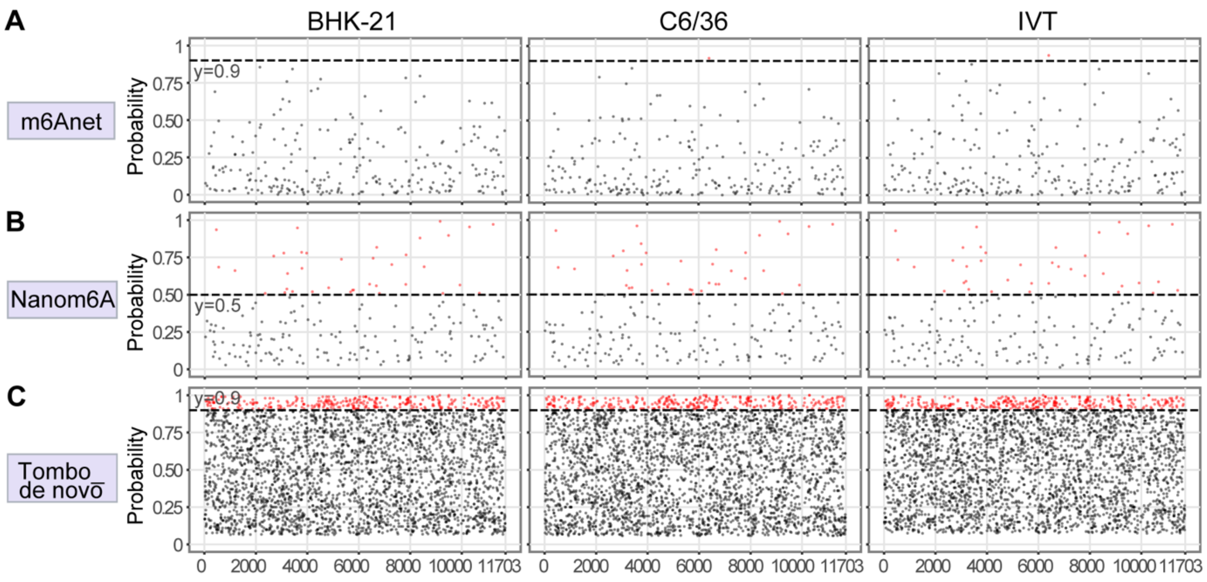

Figure S3 Prediction of m6A sites in different samples using single-mode tools. (A), (B), and (C) Outputs for m6A mapping in different samples using m6Anet, Nanom6A, and Tombo_de novo, respectively. The dashed lines indicate empirical cutoff values. Potential m6A positions are labeled in red.

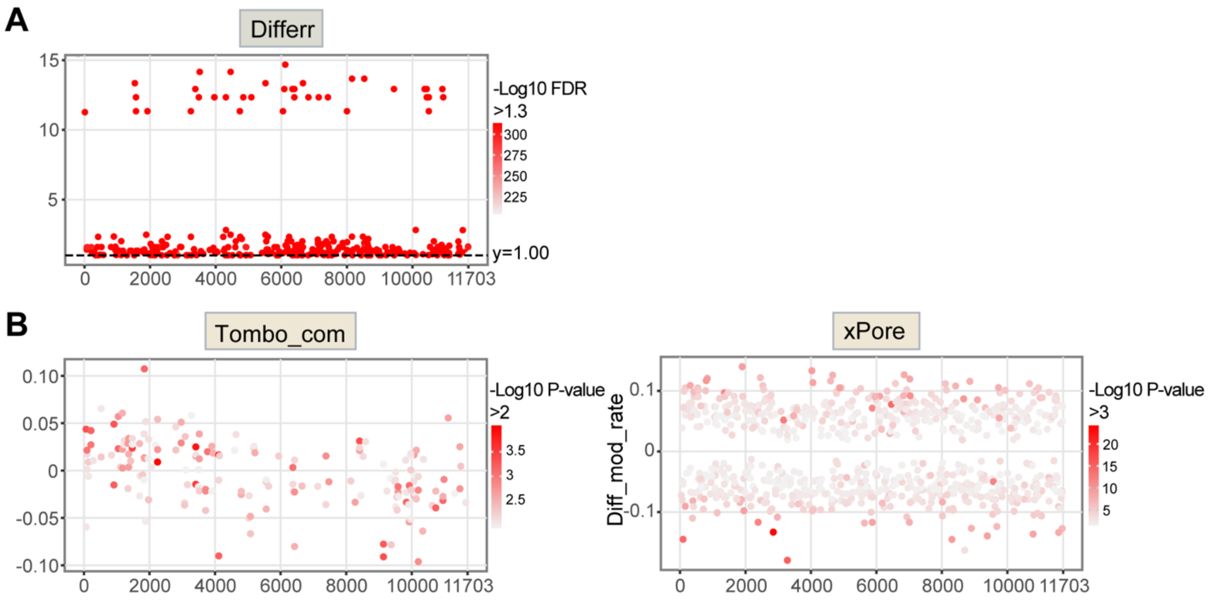

Figure S4 Predictions of RNA modifications in the IVT sample using comparative tools. IVT reads were divided into two subgroups and used as input data for comparison. Default or recommended thresholds were applied. All detected modifications are included. (A) and (B) False positive predictions of RNA modifications in the IVT sample by error rate-based computational tools Differr or by current signal-based computational tools Tombo_com and xPore.

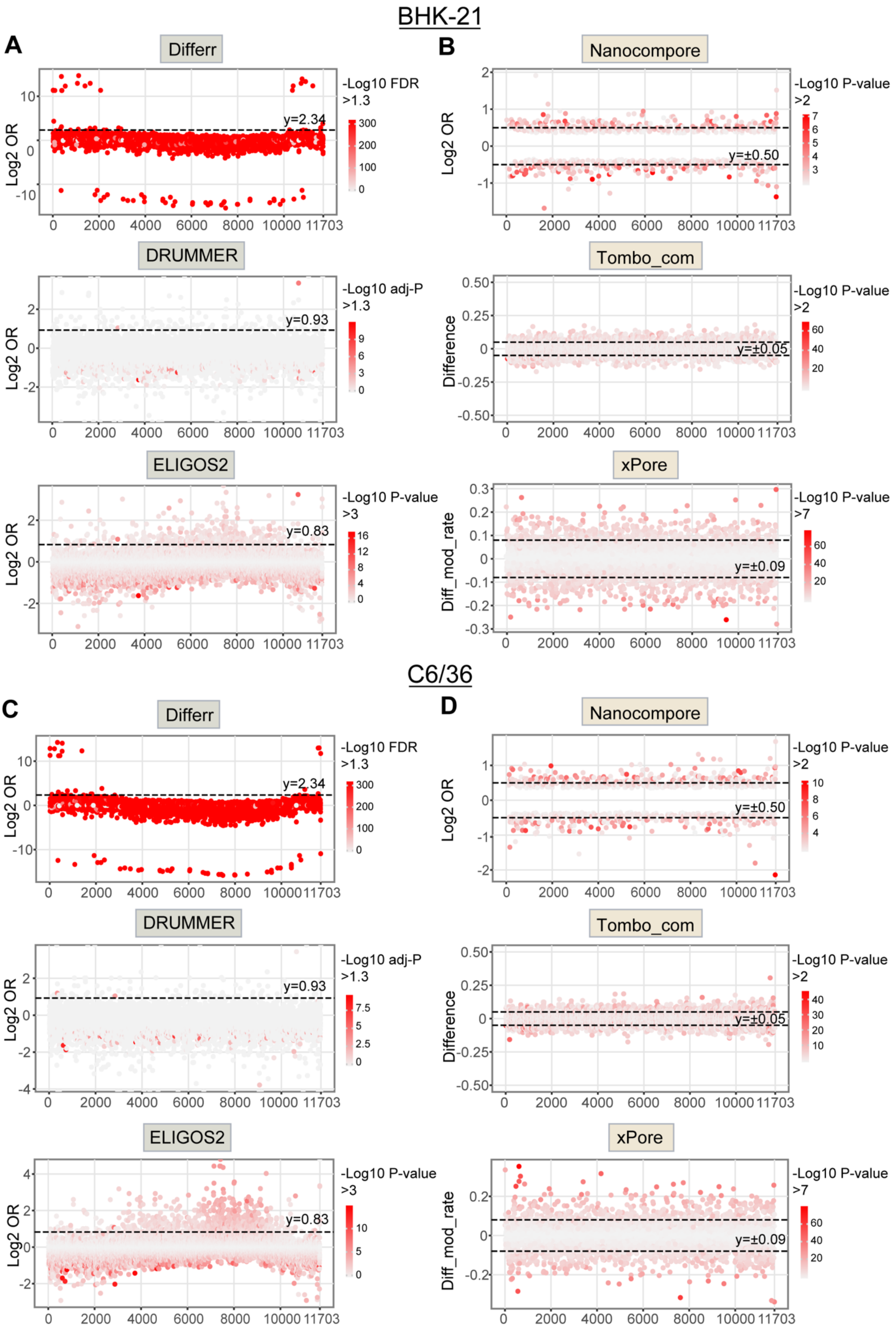
BHK-21
A
B
Differr
-Log10 FDR >1.3
y=2.34
Log2 OR
Nanocompore
-Log10 P-value >2
y=±0.50
DRUMMER
-Log10 adj-P >1.3
y=0.93
Tombo_com
Difference
y=±0.05
ELIGOS2
-Log10 P-value >3
y=0.83
xPore
-Log10 P-value >7
Diff_mod_rate
y=±0.09
C6/36
C
D
Differr
-Log10 FDR >1.3
y=2.34
Nanocompore
-Log10 P-value >2
y=±0.50
DRUMMER
-Log10 adj-P >1.3
y=0.93
Tombo_com
y=±0.05
ELIGOS2
-Log10 P-value >3
y=0.83
xPore
-Log10 P-value >7
y=±0.09
0 2000 4000 6000 8000 10000 11703

Figure S5 Identification of modification sites in the SINV genome using comparative tools. An equal number of reads (772 in amount) were subsampled from the BHK-21, C6/36, and IVT full-length viral reads. Comparisons were performed between BHK-21 and IVT reads or between C6/36 and IVT reads. Adjusted cutoff values were applied accordingly. All detected modifications are included. (A) and (B) Predictions of modification sites in the SINV genome produced in BHK-21 cells using error- or signal-based comparative methods. (C) and (D) Predictions of modification sites in the SINV genome produced in C6/36 cells using error- or signal-based comparative methods.

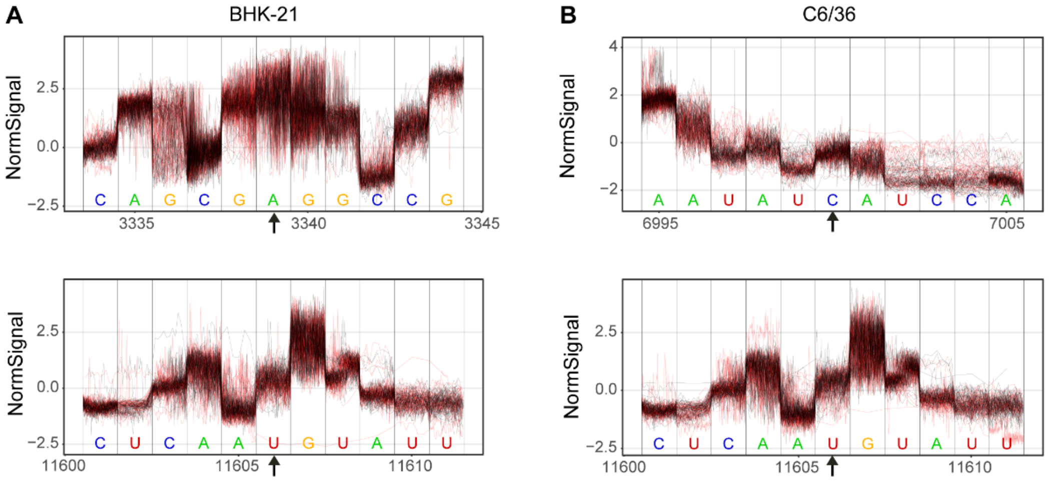

Figure S6 Raw signal traces at predicted modification sites and adjacent regions, corresponding to Figures 4E and F. Black arrows indicate the locations of predicted modification sites.

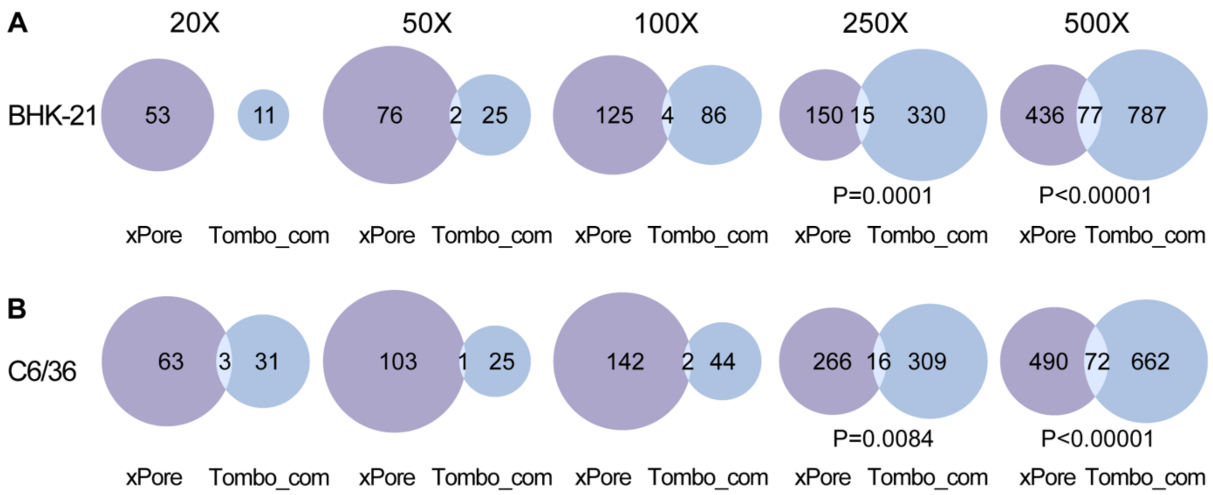

Figure S7 Intersections of the outputs from Tombo_com and xPore analyses at different coverage depths with corresponding optimized cutoff values. Native and IVT viral reads were subsampled to certain equivalences, followed by analyses using Tombo_com and xPore with corresponding optimized cutoff values. The intersections of Tombo_com and xPore at individual coverage depths are presented. (A) Predictions of SINV RNAs generated in BHK-21 cells. (B) Prediction of SINV RNAs generated in C6/36 cells. P-values of Fisher's exact tests are indicated accordingly.

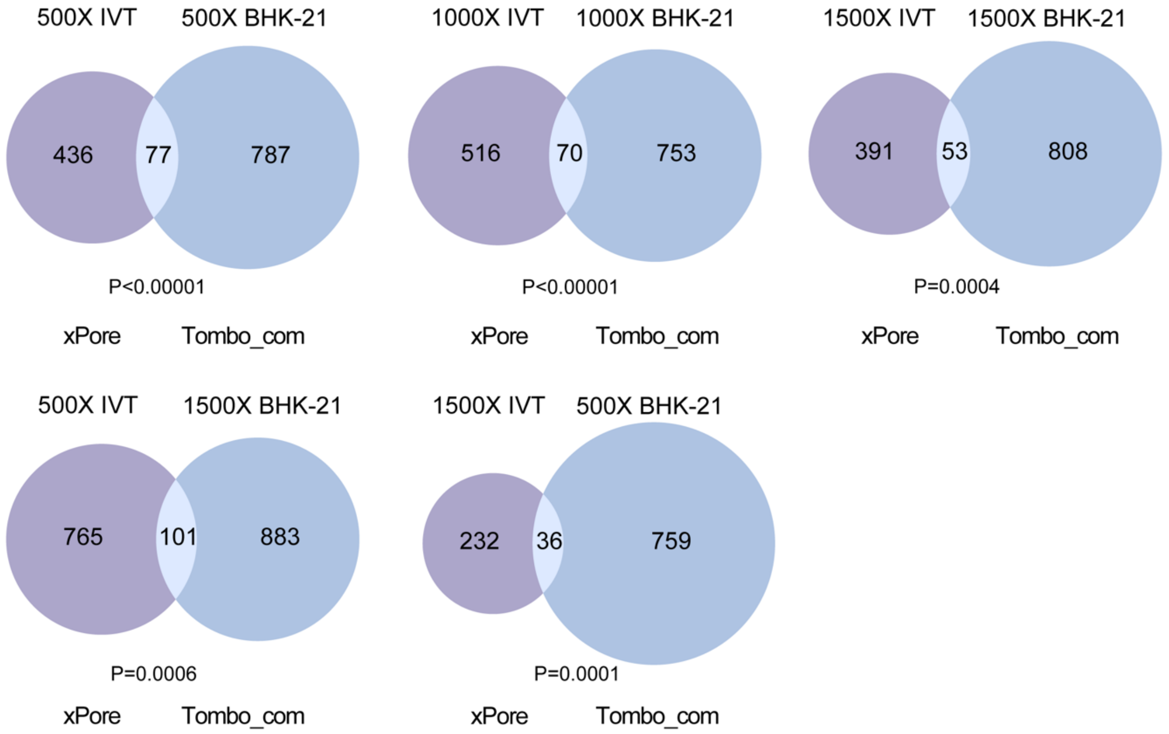

Figure S8 Intersections of the outputs from Tombo_com and xPore analyses at different coverage depths using the optimized cutoffs at the coverage depth of 500. BHK-21 native and IVT viral reads were subsampled to certain equivalences, followed by analyses using Tombo_com and xPore with the optimized cutoffs at the coverage depth of 500. P-values of Fisher's exact tests are indicated accordingly.
